# Prokaryotic expression, polyclonal antibody production, and application of yak TGF-β2

**DOI:** 10.1590/1984-3143-AR2025-0006

**Published:** 2025-10-24

**Authors:** Yaming Chen, Yangyang Pan, Sijiu Yu, Jinglei Wang, Jiangfeng Fan

**Affiliations:** 1 College of Veterinary Medicine, Gansu Agricultural University, Lanzhou, Gansu, China; 2 The First School of Clinical Medical, Gansu University of Chinese Medicine, Lanzhou, Gansu, China; 3 Technology and Research Center of Gansu, Lanzhou, Gansu, China

**Keywords:** yak, TGF-β2, prokaryotic expression, polyclonal antibody

## Abstract

This study aimed to generate yak-specific polyclonal antibodies against transforming growth factor beta 2 (TGF-β2). Specific primers targeting the TGF-β2 coding sequence (CDS) were designed, and the gene was amplified via RT-PCR. The amplified product was cloned into the pET-32a(+) vector to construct the recombinant plasmid pET-32a(+)-TGF-β2. This plasmid was transformed into Escherichia coli BL21(DE3) for protein expression. Isopropyl β-D-1-thiogalactopyranoside (IPTG) induced TGF-β2 production, and the recombinant protein was purified. New Zealand rabbits were immunized with the purified protein to generate polyclonal antibodies. Polyclonal antibody titers were determined using ELISA, while specificity was assessed through Western blot and immunohistochemistry. The recombinant plasmid was successfully constructed, and IPTG induction yielded a 63 kDa protein. Optimal expression occurred at 25 °C with 0.5 mmol·L^−1^ IPTG and a 10-hour induction period. ELISA confirmed an antibody titer of 1:10^6^. Western blot and immunohistochemistry demonstrated TGF-β2 expression in female yak ovaries, oviducts, and uteri across reproductive stages, with significantly elevated ovarian levels during pregnancy. This study successfully produced and validated a highly specific anti-yak TGF-β2 polyclonal antibody, providing a vital tool for investigating its role in yak reproductive physiology.

## Introduction

The TGF-β superfamily comprises essential peptide growth factors that regulate critical biological processes, including cell proliferation, differentiation, adhesion, apoptosis, extracellular matrix formation, and embryonic development (Xie et al., 2014). Members of this superfamily, such as TGF-βs, activins, growth differentiation factors, inhibins, and bone morphogenetic proteins, form stable dimers through disulfide bonds. Disruption of these bonds releases active molecules, enabling their biological functions (Huminiecki et al., 2009). Among these, TGF-β family members are particularly important in animal reproduction (Knight and Glister, 2006). TGF-β2, a key member of the TGF-β superfamily, plays an essential role in mammalian reproductive growth and development by contributing to processes such as osteoblast differentiation, fibrosis, and reproduction (Huai et al., 2022; Huang and Zhang, 2022; Li et al., 2022). Studies in other species have demonstrated its regulatory effects on oocyte growth and development in tilapia, fatty acid and glucose metabolism, pregnancy stability, and follicular growth and degeneration in rat ovarian tissue (Long, 2019; Ergin et al., 2008; Jovanović and Kramer, 2010; Takahashi et al., 2019). Additionally, TGF-β2 is broadly expressed during chicken embryonic development, across all three germ layers (Sundaresan et al., 2008; Yamagishi et al., 2012), and its expression in human retained miscarriage tissue suggests a role in embryo implantation and miscarriage prevention (Wang et al., 2021). Collectively, these findings underscore TGF-β2’s pivotal role in reproductive development across species.

Yaks, indigenous to the high-altitude regions of China, are renowned for their resilience to extreme environments, strength, and ability to endure cold and fatigue, earning them the nickname “Boat of the Plateau” (Wang, 2017). As a crucial component of Tibetan Plateau agriculture, they provide essential economic support to local herding communities. While TGF-β2 has been extensively studied in mammalian reproduction, its functional significance in yaks—particularly in the context of high-altitude adaptation—remains poorly understood. This knowledge gap is further compounded by the absence of species-specific immunological tools, despite the likely pivotal role of TGF-β2 in yak reproductive resilience. Our study addresses this critical need by developing targeted antibodies against yak TGF-β2, which will enable detailed investigations into its regulatory mechanisms under extreme plateau conditions.

This study sought to bridge this knowledge gap by cloning the coding sequence (CDS) of the yak TGF-β2 gene, constructing a prokaryotic expression vector, and expressing a fusion protein. The purified protein was used to immunize New Zealand rabbits to produce TGF-β2 polyclonal antibodies. The efficacy and specificity of these antibodies were assessed, laying a theoretical foundation for further research into the role of TGF-β2 in yak reproduction and its regulatory mechanisms.

## Methods

### Ethics statement

All animal experiments were approved by the Ethies Committee of Gansu Agricultural University,2022(GSAU-Eth-VMC-2022-23).

### Experimental materials

Yak uterine samples were collected in May 2023 from a slaughterhouse in Xining, Qinghai Province. Competent *E. coli* strains DH5α and BL21 (DE3) were obtained from Beijing Qinke Xinye Biotechnology Co., Ltd. DNA Marker (DL 2000), Taq polymerase, PCR reagents, and DNA purification kits were sourced from TaKaRa Bioengineering Co., Ltd. The PET-32a(+) expression vector, restriction enzymes BamHI and XhoI, and T4 DNA ligase were all purchased from TaKaRa. Nickel columns were supplied by GE Healthcare, and IPTG was procured from Beijing Tiangen Biochemical Technology Co., Ltd.

### Primer design and synthesis

Primers were designed using Primer Premier 6 software, incorporating BamHI and XhoI restriction sites at both ends to amplify a 1197 bp product. The sequences were: upstream primer, CTTTTGCCGACTCCCTCA; downstream primer, CGTTTCAGATGCCAGTTTTA. All primers were synthesized by Sangon Biotech C., Ltd.

### RNA extraction and cDNA synthesis

RNA was extracted from tissue samples using TRIzol reagent. RNA quality was verified by spectrophotometry (A260/280>1.8) and capillary electrophoresis (RIN≥7).The extracted RNA was reverse-transcribed into cDNA, which was subsequently stored at -20°C.

### PCR amplification of the TGF-β2 gene

The PCR reaction mixture consisted of 1 μL of cDNA template, 1 μL of each primer, 12.5 μL of Taq PCR Master Mix, and 9.5 μL of ddH_2_O, for a final volume of 25 μL. The PCR protocol included an initial denaturation step at 95°C for 4 minutes, followed by 35 cycles of 95°C for 30 seconds, 60°C for 30 seconds, and 72°C for 15 seconds. The process concluded with a final extension at 72°C for 5 minutes. PCR products were separated on a 1% agarose gel, and the desired fragments were purified.

### Construction of the prokaryotic expression vector

The DNA fragment generated by restriction digestion was inserted into the pET-32a vector using BamHI and XhoI enzymes. Ligation was performed overnight at 16°C with T4 DNA ligase. The recombinant plasmid was transformed into DH5ɑ competent cells, plated on LB agar with ampicillin, and incubated at 37°C overnight. Positive clones were selected, and plasmid DNA was extracted. Verification was conducted by PCR, restriction digestion, and sequencing (Sangon Biotech). The verified plasmid was subsequently transformed into BL21(DE3)/Rosetta competent cells for further study.

### Expression of TGF-β2 recombinant protein

The recombinant plasmid pET-32a-TGF-β2 was transformed into BL21(DE3) competent cells. Single colonies were selected and cultured overnight at 37°C with shaking. The bacterial suspension was incubated at 37°C, and IPTG was added after 2–3 hours to induce protein expression overnight. After 30 hours, cells were harvested, lysed using an ultrasonic disruptor, and analyzed by SDS-PAGE.

### Optimization of TGF-β2 recombinant protein expression conditions

Optimization of Inducer Concentration. The TGF-β2 recombinant bacteria were cultured in 5 mL of TB medium for 16 hours. When the optical density reached 0.6–0.8, various inducer concentrations (0.2, 0.5, 0.7, 1, and 1.5 mmol·L^-1^) were tested at 37°C. After 6 hours of induction, 1 mL from each culture was analyzed by SDS-PAGE to determine the optimal inducer concentration for protein expression.

Optimization of Induction Temperature. The TGF-β2 recombinant bacteria were cultured in 5 mL of TB medium at 37°C. When the OD reached 0.6–0.8 after 16 hours, 300 mmol·L^-1^ IPTG was added. Cultures were induced at 16°C, 20°C, 25°C, 30°C, and 37°C with shaking. One-milliliter samples were collected post-induction, and SDS-PAGE was performed to determine the optimal induction temperature.

Optimization of Induction Time. The TGF-β2 recombinant bacteria were cultured in 5 mL of TB medium at 37°C with shaking. After 16 hours, when the optical density (OD) reached 0.6–0.8, induction was initiated by the addition of 300 mmol·L^-1^ IPTG at 37°C. Samples were collected at 2, 4, 6, 8, 10, 16, 20, and 24 hours and subjected to SDS-PAGE analysis to determine the optimal induction time for protein expression.

### Recombinant protein purification

Positive clones were cultivated under optimized induction conditions, and harvested cells were resuspended in PBS buffer. Cell lysis was performed by sonication on ice at 420 W, using 3-second pulses with 5-second intervals, for 15 minutes. The lysate was centrifuged at 12,000 rpm and 4°C for 20 minutes to separate the supernatant and pellet. Recombinant protein was purified from the denatured fractions using the Ni-NTA affinity chromatography protocol, and purification efficiency was assessed by SDS-PAGE.

### Preparation and characterization of polyclonal antibodies

The TGF-β2 recombinant protein was emulsified with Freund’s complete adjuvant in a 1:1 ratio. New Zealand rabbits were subcutaneously injected with an initial dose of 0.5 mg per rabbit at multiple sites. Weekly booster doses of 0.25 mg per rabbit, prepared with Freund’s incomplete adjuvant, were administered from weeks 2 to 5. Antibody titers were assessed one week after the final injection. Serum from rabbits with stable antibody expression was collected and stored at -80°C. Western blot confirmed antibody titers, and the antibodies were used to evaluate TGF-β2 expression in ovarian, uterine, and oviduct tissues.

### Determination of polyclonal antibody titers

Antibody titers were assessed via indirect ELISA. TGF-β2 recombinant protein, diluted to 1.0 μg/mL, was used as the antigen to coat 96-well ELISA plates (100 μL per well), with negative and blank controls included. Plates were incubated overnight at 4°C. After three PBST washes, wells were blocked with 5% skim milk at 37°C for 3 hours and washed three additional times. Diluted rabbit polyclonal serum (100 μL per well) was added, while negative control wells received negative serum. Following a 2-hour incubation at 37°C and three washes, 100 μL of HRP-conjugated goat anti-rabbit IgG was introduced. After a 1-hour incubation at 37°C and five washes, 100 μL of TMB substrate was added and incubated for 10 minutes at 37°C. The reaction was stopped with 50 μL of 2 mol/L H_2_SO_4_, and absorbance was measured at 450 nm using a microplate reader.

### Applications of Yak TGF-β2 polyclonal antibody

Western Blot Analysis of Yak TGF-β2 Polyclonal Antibody Expression in Reproductive Organs of Female Yaks Across Different Reproductive Phases

Ovarian, uterine, and oviduct tissues were collected from 3–6-year-old parous female yaks during pregnancy, follicular, and luteal phases. Total protein was extracted, quantified using a BCA protein assay kit (DQ111-01, Full Gold, Beijing), and separated by SDS-PAGE. The proteins were transferred to PVDF membranes (YA1701, Solarbio, Beijing), blocked with 5% skim milk (D8340, Solarbio, Beijing) at 37°C for 2 hours, and incubated overnight at 4°C with primary antibodies: TGF-β2 (1:5000) and GAPDH (1:2500, Abcam, USA). After a 1-hour wash with TBST, membranes were incubated with HRP-conjugated goat anti-rabbit IgG (1:5000, Abcam, USA) for 2 hours, followed by a 1.5-hour wash. Protein bands were visualized using ECL and quantified with ImageJ, with GAPDH as the internal control. All experiments were performed in triplicate.

Immunohistochemical Analysis of Yak TGF-β2 Polyclonal Antibody Expression in Reproductive Organs of Female Yaks Across Different Reproductive Phases

Ovarian, uterine, and oviduct tissues from 3–6-year-old parous female yaks during pregnancy, follicular, and luteal phases were fixed in 4% paraformaldehyde and processed through ethanol dehydration and xylene clearing before embedding in paraffin. Paraffin blocks were sectioned into 4 μm slices, underwent antigen retrieval and blocking (Zhang, 2021), and incubated overnight at 4°C with TGF-β2 polyclonal antibody (1:4000). Negative controls were treated with PBS. After three PBS washes (3 minutes each), sections were incubated with biotinylated goat anti-rabbit IgG (SPA kit solution B) at 37°C for 15 minutes, followed by HRP-conjugated streptavidin (SPA kit solution C) at room temperature for 15 minutes. After three additional PBS washes, DAB substrate was applied for color development. Sections were counterstained with hematoxylin, dehydrated, cleared, and mounted. Sections were examined and photographed under a microscope.

### Data analysis

A one-way ANOVA was performed to analyze TGF-β2 protein expression levels using SPSS 25.0, with three replicates per group. Results are presented as the mean ± standard error, with statistical significance set at P < 0.05. Graphs were generated using GraphPad Prism 8.

## Results

### Construction of the pET-32a(+)-TGF-β2 prokaryotic expression vector

**Figure 1:**
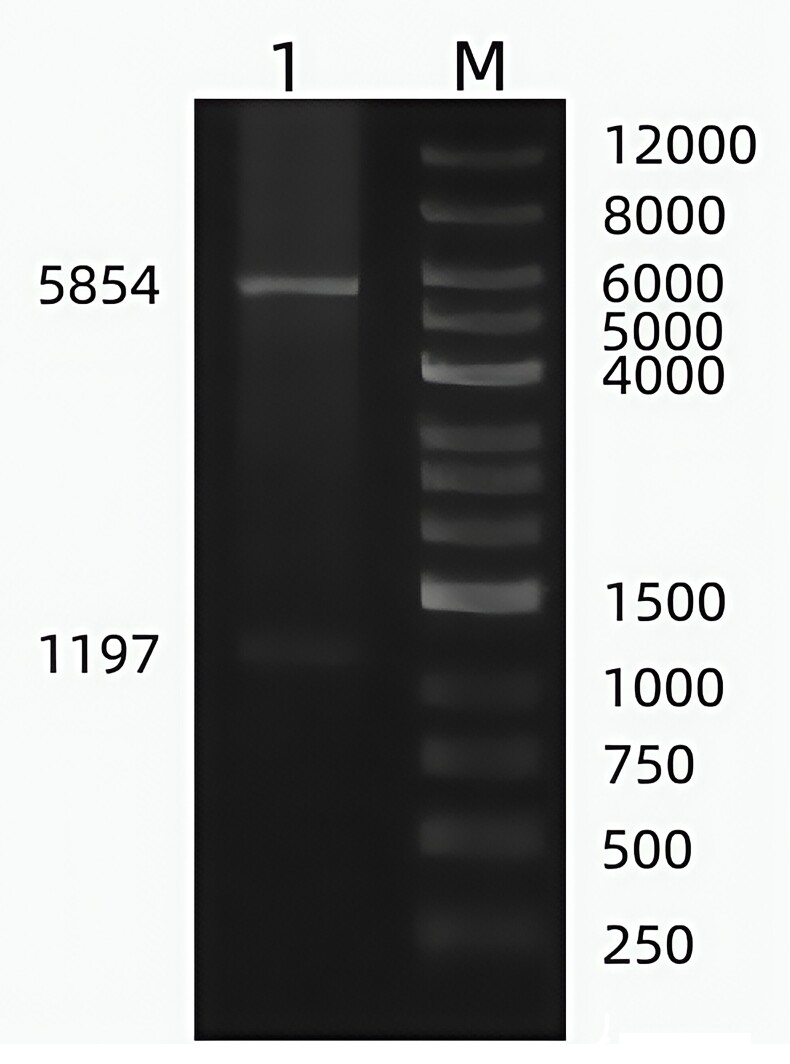
PCR identification of the recombinant prokaryotic expression vector pET-32a-TGF-β2 after double digestion. M: DL12000 DNA Marker; 1: BamHI and XhoI double digestion of pET-32a(+)-TGF-β2 plasmid product.

The target gene was successfully inserted into the pET-32a(+) vector. The recombinant plasmid, pET-32a(+)-TGF-β2, was confirmed by double enzyme digestion, which yielded two bands: a 1197 bp fragment and the pET-32a(+) vector band (Figure 1). Sequencing analysis verified that the positive clones matched the TGF-β2 gene sequence from GenBank, confirming the successful construction of the recombinant plasmid.

### Induced expression of TGF-β2 protein in different clonal strains

**Figure 2:**
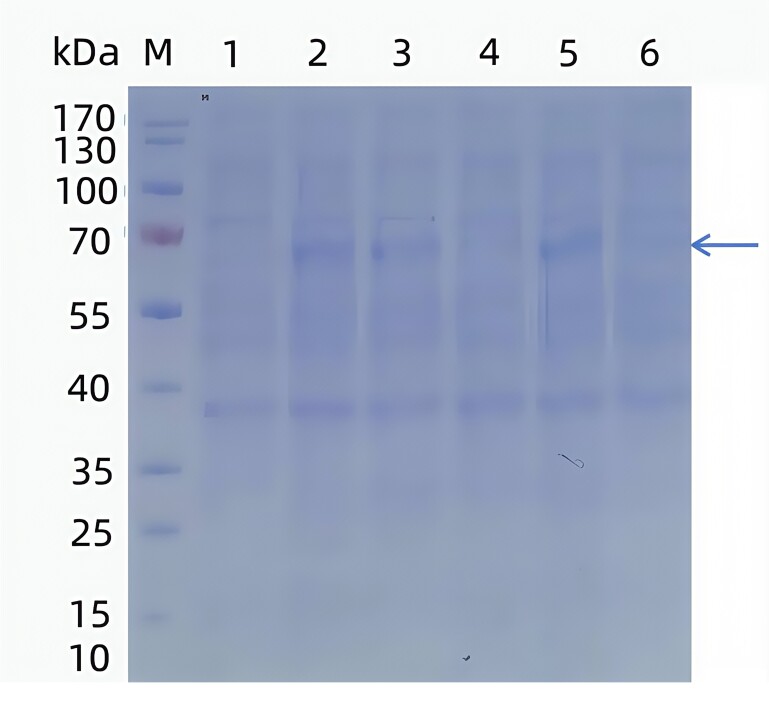
SDS-PAGE results of TGF-β2 protein induced expression in different monoclonal strains. M. Protein Marker; 1: Strain without IPTG induction; 2: Strain with IPTG induction.

The recombinant plasmid pET-32a(+)-TGF-β2 was transformed into BL21 competent cells, and protein expression was induced with IPTG. SDS-PAGE analysis demonstrated a strong 63 kD band in lanes 2, 3, and 5, consistent with the expected size of the expressed protein (Figure 2). Weaker 63 kD bands were observed in lanes 4 and 6, whereas lane 1, without IPTG induction, showed no detectable protein bands.

### Optimization of TGF-β2 recombinant protein induction conditions

**Figure 3:**
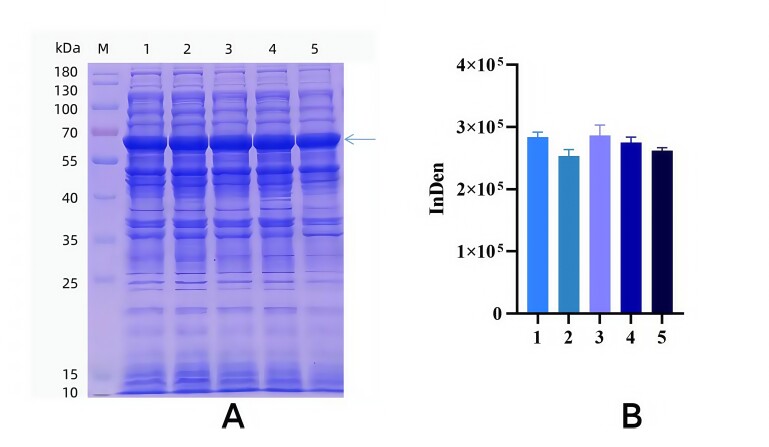
Optimization of IPTG concentration for recombinant TGF-β2 expression. A. Optimization of IPTG concentration of TGF-β2 recombinant protein; M. Protein Marker; 1-6 represent the level of IPTG are 0,0.2,0.5,0.7,1 and 1.5 mmol·L^-1^, respectively. B. Gray value analysis results of different inducer concentrations.

Optimal Inducer Concentration. Cultures were incubated at 37°C for 3 hours before IPTG was added at final concentrations of 0.2, 0.5, 0.7, 1.0, and 1.5 mmol·L^−1^ for overnight induction. SDS-PAGE analysis demonstrated that protein expression peaked at 1.5 mmol·L^−1^ IPTG (Figure 3). Thus, 1.5 mmol·L^−1^ was determined to be the optimal inducer concentration for recombinant protein expression.

**Figure 4:**
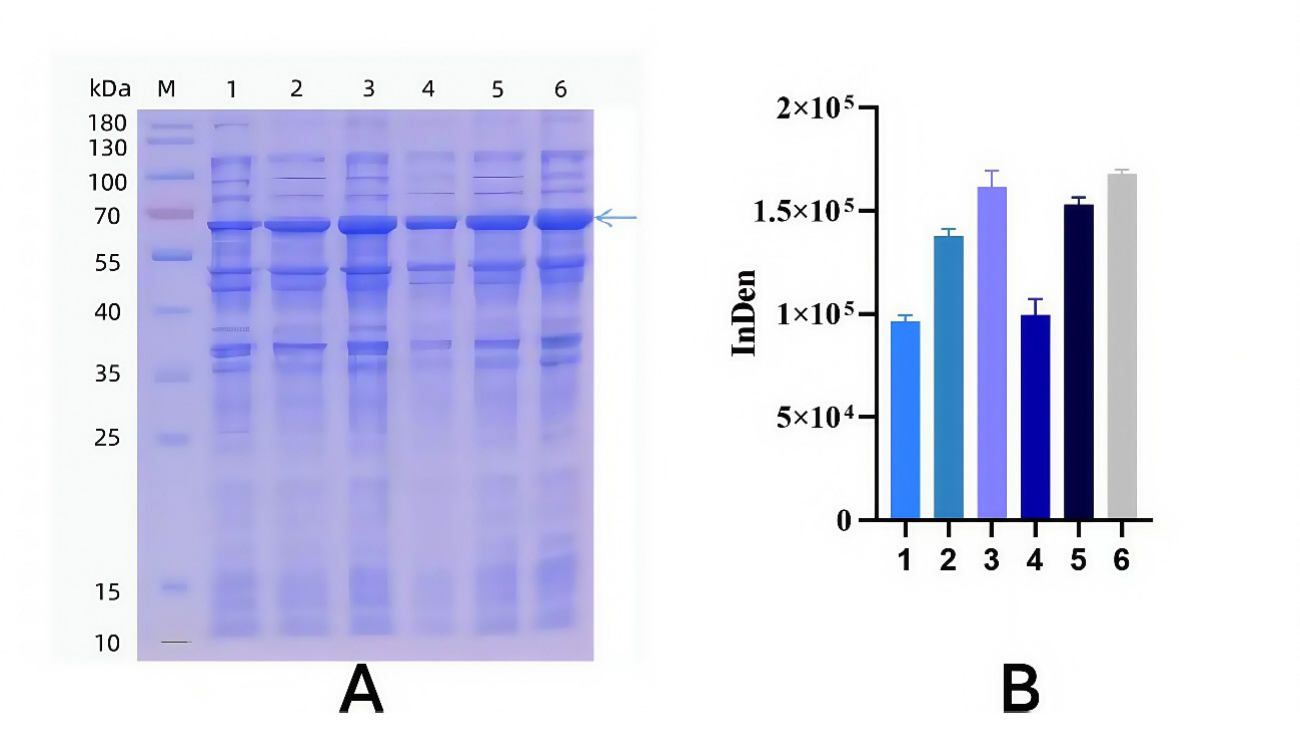
Optimum temperature selection for inducing TGF-β2 recombinant protein. A. Optimum temperature selection for inducing TGF-β2 recombinant protein; M. Protein Marker; 1-5 represent the induced temperature are 16,20,25,30 and 37 ℃, respectively. B. Different temperatures induced SDS-PAGE gray value analysis results.

Optimal Induction Temperature. Cultures were incubated at 37°C for 3 hours before overnight induction with 0.5 mmol·L^−1^ IPTG at 16, 20, 25, 30, and 37°C. SDS-PAGE analysis demonstrated that recombinant protein expression was maximized at 25°C (Figure 4). Accordingly, 25°C was determined to be the optimal induction temperature for recombinant protein production.

**Figure 5:**
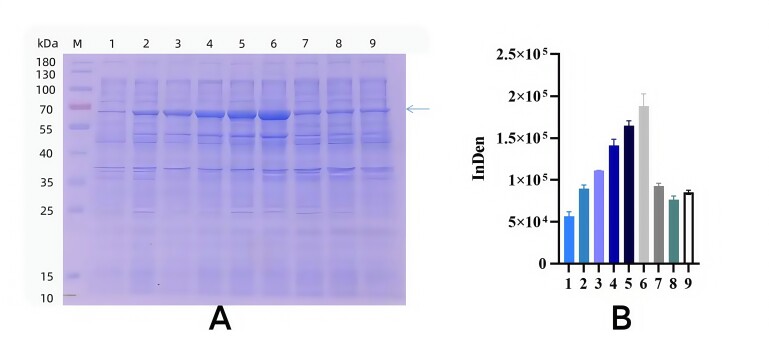
Optimization of induction time of TGF-β2 recombinant protein. A. Optimization of induction time of yak EPF recombinant protein; M. Protein Marker; 1. Negative control; 2-8 represent the induction time are2,4,6,8,10,14,18,22 h, respectively. B. Induced SDS-PAGE gray value analysis results at different times.

Optimal Induction Time. Cultures were incubated at 25°C for 3 hours before the addition of 0.5 mmol·L^−1^ IPTG. Induction was performed at 25°C, with samples collected at 2–4 hour intervals over a 22-hour period and stored at 4°C. SDS-PAGE analysis demonstrated that yak TGF-β2 recombinant protein expression peaked at 10 hours (Figure 5A). Gray scale analysis further confirmed variations in expression levels across induction times (Figure 5B). Accordingly, 10 hours was determined to be the optimal induction time for recombinant protein expression.

### Induction and Purification of recombinant TGF-β2 protein

**Figure 6:**
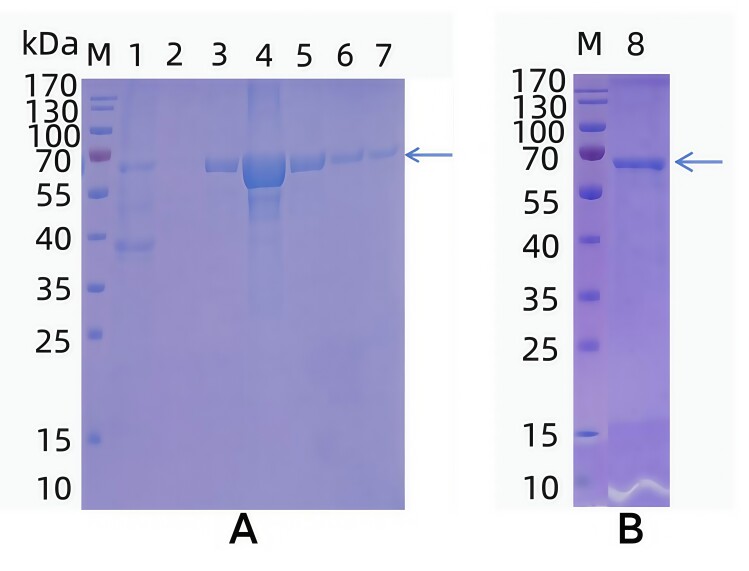
Expression and purification of TGF-β2 recombinant protein. A. SDS-PAGE analysis of purified TGF-β2 recombinant protein. B. Results of SDS-PAGE analysis of TGF-β2 recombinant protein after dialysis. M.Protein Marker; 1:Flow-through; 2:Equilibration buffer; 3:5 mmol·L^-1^ eluent; 4:20 mmol·L^-1^ eluent; 5:50 mmol·L^-1^ eluent; 6:80 mmol·L^-1^ eluent; 7:300 mmol·L^-1^ eluent; 8:Purified recombinant TGF-β2 protein after dialysis.

The recombinant TGF-β2 protein was purified using a Ni column and eluted with a gradient of buffers. Lower buffer concentrations effectively removed nonspecific proteins, whereas higher concentrations (5, 50, 80, and 300 mmol·L^−1^) yielded minimal amounts of the target protein. Optimal recovery was achieved at 20 mmol·L^−1^, ensuring efficient purification (Figure 6A). Following dialysis, SDS-PAGE analysis verified that the purified TGF-β2 protein was free of nonspecific contamination (Figure 6B).

### Serum antibody titer of fusion protein TGF-β2

**Figure 7:**
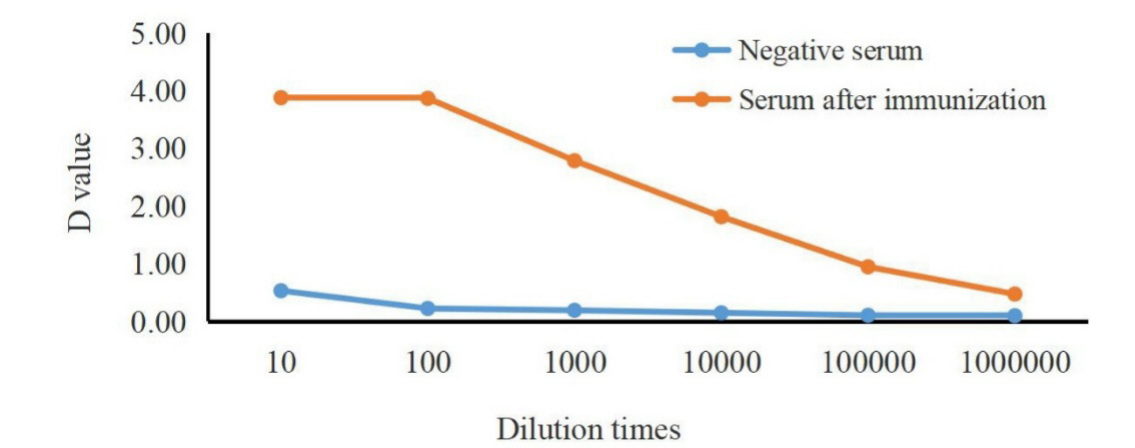
Antibody titer of TGF-β2 antiserum.

**Figure 8:**
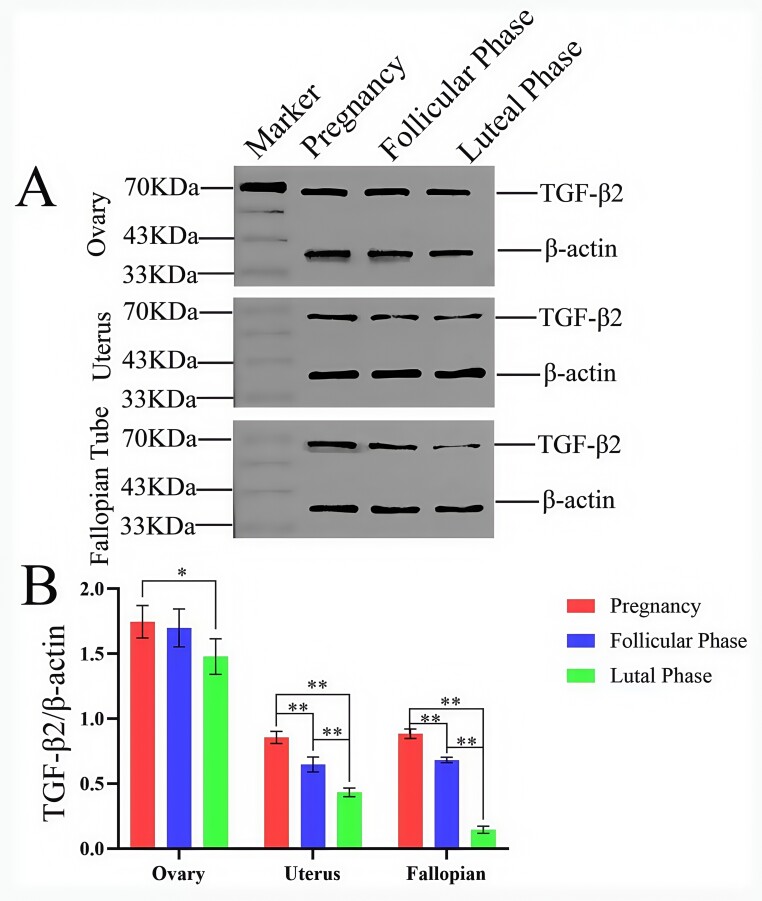
TGF-β2 Protein Expression in Female Yak Reproductive Organs Across Different Reproductive Stages. A. Western blot showing TGF-β2 protein expression in female yak reproductive organs across various reproductive stages; B. Gray scale analysis of TGF-β2 protein expression levels in different yak tissues. (* denotes significant intergroup differences, P < 0.05; ** denotes highly significant differences, P < 0.01).

The serum antibody titer of New Zealand rabbits immunized with the purified TGF-β2 fusion protein was measured by ELISA. At a dilution of 1:10^6^, the OD450 of the positive serum exceeded that of the negative serum by more than 2.1-fold, yielding an antibody titer of 1:10^6^ (Figure 7). These findings indicate that the prokaryotic TGF-β2 fusion protein elicited a robust immune response in New Zealand rabbits. Western blot analysis (Figure 8) confirmed TGF-β2 protein expression in yak ovary, oviduct, and uterus tissues, with a distinct band observed at ~63 kDa, verifying the strong specificity and reactivity of the polyclonal antibody.

### Analysis of TGF-β2 polyclonal antibody expression in yak reproductive organs using western blot during different reproductive phases

Western blot analysis (Figure 8) showed that ovarian TGF-β2 levels during pregnancy were significantly higher than those observed during the luteal phase (P < 0.05), but no significant difference was detected when compared to the follicular phase (P > 0.05). TGF-β2 expression in the uterus and oviduct during pregnancy was significantly higher than in both the follicular and luteal phases (P < 0.01). Furthermore, expression levels during the follicular phase were significantly elevated compared to those in the luteal phase (P < 0.01).

### TGF-β2 polyclonal antibody expression in female yak reproductive organs during different reproductive phases

**Figure 9:**
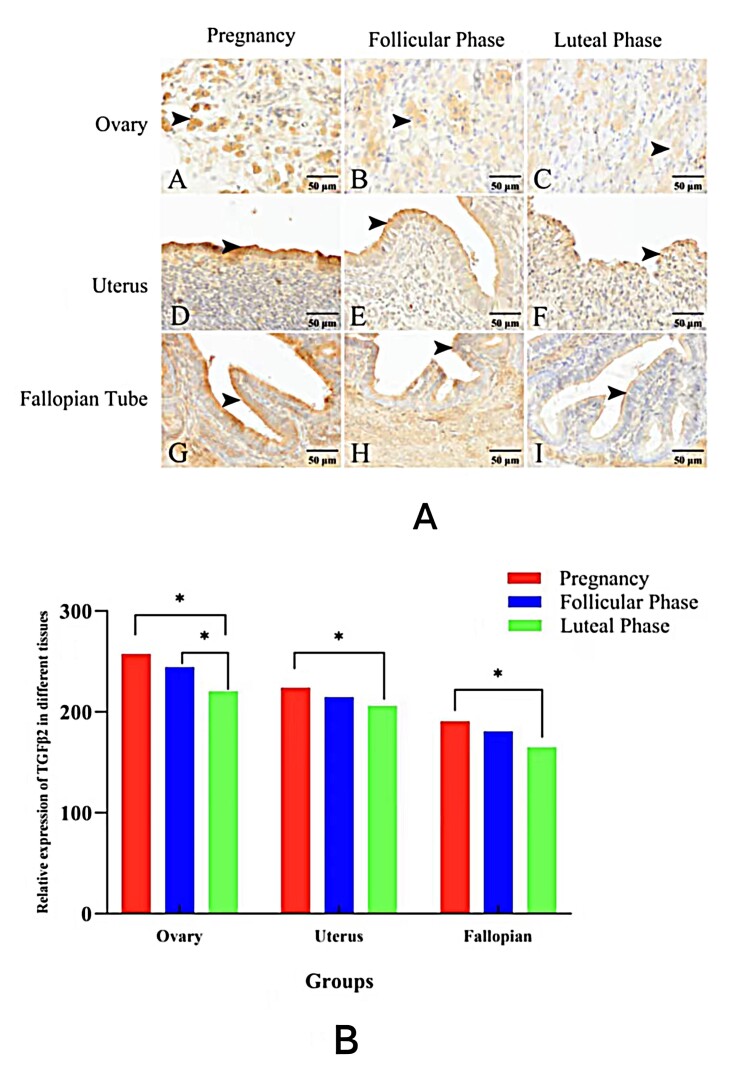
TGF-β2 Protein Distribution in Reproductive Organs of Female Yaks During Different Reproductive Stages. A-C. TGF-β2 protein distribution in the ovary across the pregnancy, follicular, and luteal phases; D-F. TGF-β2 protein distribution in the oviduct during the pregnancy, follicular, and luteal phases; G-I. TGF-β2 protein distribution in the uterus across the pregnancy, follicular, and luteal phases; (* indicates statistically significant differences between groups, P < 0.05; ** indicates highly significant differences, P < 0.01).

Immunohistochemical analysis revealed that TGF-β2 expression in ovarian granulosa cells was significantly higher during the pregnancy and follicular phases compared to the luteal phase (P < 0.05) (Figures 9A–C). In the oviduct and uterus, TGF-β2 levels were significantly elevated during pregnancy relative to the luteal phase (P < 0.05).

## Discussion

TGF-β2, a key member of the TGF-β superfamily, is a secreted protein that regulates diverse biological processes, including growth and reproduction (Wang et al., 2023b). Previous studies from our group demonstrated that TGF-β2 contributes to the growth of yak hair follicles and sweat glands. Moreover, TGF-β2 plays a critical role across species, from insects to mammals, in modulating the growth and differentiation of somatic and germ cells within the reproductive system and gonads. This factor also governs cyclical changes in the endometrium, establishing a microenvironment conducive to embryo implantation and pregnancy maintenance. During gonadal development, TGF-β2 binds to specific receptors to activate downstream signaling pathways—both SMAD-dependent and SMAD-independent—thereby coordinating gonadal cell proliferation, differentiation, and apoptosis (Liu et al., 2022; Monsivais et al., 2017). Consequently, the development of effective antibodies against TGF-β2 is of paramount importance for further elucidating its functions.

This study successfully generated a specific rabbit anti-yak TGF-β2 polyclonal antibody using prokaryotic expression and animal immunization. Stable recombinant protein production is fundamental for effective polyclonal antibody generation (Gu et al., 2021). However, prokaryotic expression of complex proteins like TGF-β2 in E. coli can be challenging. Critical determinants include the expression vector, host strain, and induction conditions, with the vector and host exerting the most significant influence (Kondo and Yumura, 2020). We selected the pET system, recognized for its reliability in cloning and expressing foreign genes in *E. coli* (Zare et al., 2019). This system utilizes the highly active and specific T7 RNA polymerase to drive target gene expression under T7 promoter control (Soman et al., 2018). Among tested vectors, pET-32a(+) demonstrated optimal efficacy for TGF-β2 expression. Its integrated His-tag not only enhanced recombinant protein yield and streamlined purification via affinity chromatography but also facilitated confirmation of expression. Furthermore, host strain selection is paramount. We employed BL21(DE3), a preferred *E. coli* strain for recombinant protein production due to its rapid growth in defined media, low protease activity, and suitability for high-density culture (Du et al., 2021). Existing TGF-β2 antibodies are predominantly raised against human or murine epitopes or synthetic peptides. Given the significant sequence divergence in TGF-β2 between humans/mice and yaks (Bos grunniens), such antibodies often exhibit poor cross-reactivity with the yak ortholog. Crucially, our cost-effective, yak-specific antibody demonstrates precise recognition of yak TGF-β2, confirming its high immunogenicity and specificity for this species.

TGF-β2 is a key regulator of ovarian function in mammals, modulating follicular development by influencing follicle growth and atresia. This conserved role strongly suggests that TGF-β2 similarly governs folliculogenesis in yaks (Bos grunniens). Furthermore, serum TGF-β2 concentrations reflect reproductive status across species. For instance, expression peaks during canine estrus but declines in proestrus, diestrus, and anestrus (Rybska et al., 2021), while in cattle, its expression increases progressively throughout pregnancy, remaining elevated in late gestation (Azumah et al., 2022). Our findings align with this pattern, demonstrating significantly elevated TGF-β2 expression in yak ovaries, uteri, and oviducts during pregnancy compared to follicular and luteal phases, indicative of its critical role in pregnancy maintenance and fetal development (Noyola-Martínez et al., 2024). Within the ovary, granulosa cells—essential for synthesizing steroid hormones and maintaining the female reproductive cycle (Constable et al., 2024)—are key sites of TGF-β2 action. Indeed, TGF-β2 modulates estrogen production in granulosa cells by regulating key steroidogenic enzymes (Islam, 2024). Following corpus luteum formation, TGF-β2 further contributes to reproductive success by regulating progesterone synthesis and ensuring consistent secretion, thereby establishing the endocrine environment necessary for embryo implantation and early pregnancy maintenance. Our immunohistochemical analysis revealed predominant TGF-β2 expression in granulosa cells across all ovarian stages, with notably heightened levels during pregnancy, reinforcing its potential role in orchestrating estrogen and progesterone dynamics. Collectively, TGF-β2 appears crucial for maintaining ovarian endocrine balance and promoting steroid hormone secretion to support fetal development. Additionally, we observed robust TGF-β2 expression in the epithelial cells of the oviduct and uterus. This expression pattern suggests TGF-β2 regulates endometrial epithelial and stromal cell proliferation, differentiation, and extracellular matrix remodeling (Cao et al., 2022). Such actions likely contribute to establishing a microenvironment conducive to embryo adhesion and implantation, enhancing endometrial receptivity, and stimulating uterine angiogenesis—all vital processes for sustaining pregnancy progression. Consequently, TGF-β2 represents a valuable factor for investigating yak reproductive physiology, offering novel insights for enhancing reproductive performance in this species.

The quality of polyclonal antibodies is highly dependent on the induction parameters, particularly temperature and duration. In this study, optimal expression of the recombinant TGF-β2 protein was achieved at 25°C for 10 hours. Notably, these conditions differ from those reported for generating antibodies against EPF (Jiao et al., 2023), LC3B (Wang et al., 2023a), and Fas (Guo et al., 2011), suggesting that optimal induction is dictated primarily by gene-specific characteristics rather than the expression vector or host strain. The serum antibody titer against the recombinant TGF-β2 fusion protein reached 1:10^6^, significantly surpassing the 1:110,000 titer reported for a rabbit anti-human TGF-β1 polyclonal antibody.This comparative analysis highlights the enhanced reactivity and potential utility of our yak-specific antibody. In conclusion, this study successfully generated a highly specific, high-titer rabbit anti-yak TGF-β2 polyclonal antibody.

## Conclusion

This study developed a recombinant plasmid for efficient expression of yak TGF-β2 fusion protein, which demonstrated strong immunogenicity. Immunization of New Zealand rabbits with this protein yielded a high-titer, highly reactive polyclonal antibody specific for yak TGF-β2. Immunodetection confirmed the presence of TGF-β2 in female yak ovaries, oviducts, and uteri throughout all reproductive stages. The high-specificity polyclonal antibodies generated here provide an essential tool for investigating the regulatory mechanisms of TGF-β2 in yak reproductive adaptation to high-altitude environments. These findings and reagents hold significant potential for advancing yak reproductive management under plateau conditions.

## Data Availability

Research data is available in the body of the article.
